# Supplementary material for: Measuring growth, resistance, and recovery after artemisinin treatment of Plasmodium falciparum in a single semi-high-throughput assay
Source: Malar J. 2025 Aug 14;24:263. doi: 10.1186/s12936-025-05481-9 (PMC12355871; doi:10.1186/s12936-025-05481-9)
Supplement: Supplementary file 1 — Additional file 1 [file 12936_2025_5481_MOESM1_ESM.docx]

**Additional files**

| **Parasite** | ***kelch13 status*** | **Origin** | **Year Isolated** | **PC1/2** | **Publication Citation** |
| --- | --- | --- | --- | --- | --- |
| NHP4032 | wildtype | Wang Pha, Thailand-Myanmar Boarder | 2008 | 1.98 | 40, 44, 45 |
| NHP4373 | wildtype | Wang Pha, Thailand-Myanmar Boarder | 2008 | 7.1 | 40, 44, 45 |
| NHP1337 | C580Y | Mawker Thai, Thaiand-Myanmar Boarder | 2011 | 7.83 | 40, 44, 45 |
| MKK2835 | wildtype | Mae Kon Ken, Thailand-Myanmar border | 2003 | - | 40, 44, 45 |
| KH004-020-019-H9 | C580Y | Western Cambodia | 2016 | 4.98 | 50 |
| MAL31-9040-C11 | wildtype | Malawi | 2016 | - | 50 |
| VN-E10 | C580Y | Vietnam | 2016 | - |  |
| VN-C1 | C580Y | Vietnam | 2016 | - |  |
| HB3 | wildtype | Honduras | 1984 | - | 53 |
| Dd2 | wildtype | Laos | 1988 | - | 51, 52 |
| SC01 | wildtype | HB3xDd2 Unique Recombinant Progeny | 1990 | - | 47, 48, 49 |
| TC005 | wildtype | HB3xDd2 Unique Recombinant Progeny | 1990 | - | 47, 48, 49 |
| D43 | wildtype | HB3xDd2 Unique Recombinant Progeny | 1990 | - | 47, 48, 49 |
| QC34 | wildtype | HB3xDd2 Unique Recombinant Progeny | 1990 | - | 47, 48, 49 |
| 7C7 | wildtype | HB3xDd2 Unique Recombinant Progeny | 1990 | - | 47, 48, 49 |
| 7c421 | wildtype | HB3xDd2 Unique Recombinant Progeny | 1990 | - | 47, 48, 49 |
| 7c170 | wildtype | HB3xDd2 Unique Recombinant Progeny | 1990 | - | 47, 48, 49 |
| 1BB5 | wildtype | HB3xDd2 Unique Recombinant Progeny | 1990 | - | 47, 48, 49 |
| NHP4026 | wildtype | Wang Pha, Thailand-Myanmar Boarder | 2007 | 8.37 | 40, 44, 45 |
| NF54GFP | wildtype | Western Africa | 1981 | - | 46, 54 |
| AB041 | wildtype | NF54 × NHP4026 Unique Recombinant Progeny | 2019 | - | 30 |
| AB109 | wildtype | NF54 × NHP4026 Unique Recombinant Progeny | 2019 | - | 30 |
| AB118 | wildtype | NF54 × NHP4026 Unique Recombinant Progeny | 2019 | - | 30 |
| AB125 | wildtype | NF54 × NHP4026 Unique Recombinant Progeny | 2019 | - | 30 |
| AB143 | wildtype | NF54 × NHP4026 Unique Recombinant Progeny | 2019 | - | 30 |
| AB145 | wildtype | NF54 × NHP4026 Unique Recombinant Progeny | 2019 | - | 30 |
| AB168 | wildtype | NF54 × NHP4026 Unique Recombinant Progeny | 2019 | - | 30 |
| AB171 | wildtype | NF54 × NHP4026 Unique Recombinant Progeny | 2019 | - | 30 |
| AB226 | wildtype | NF54 × NHP4026 Unique Recombinant Progeny | 2019 | - | 30 |
| AB238 | wildtype | NF54 × NHP4026 Unique Recombinant Progeny | 2019 | - | 30 |
| AB239 | wildtype | NF54 × NHP4026 Unique Recombinant Progeny | 2019 | - | 30 |
| AB246 | wildtype | NF54 × NHP4026 Unique Recombinant Progeny | 2019 | - | 30 |
| AB250 | wildtype | NF54 × NHP4026 Unique Recombinant Progeny | 2019 | - | 30 |
| AB264 | wildtype | NF54 × NHP4026 Unique Recombinant Progeny | 2019 | - | 30 |
| AB277 | wildtype | NF54 × NHP4026 Unique Recombinant Progeny | 2019 | - | 30 |
| AB291 | wildtype | NF54 × NHP4026 Unique Recombinant Progeny | 2019 | - | 30 |
| AC032 | wildtype | NF54GFP × NHP4026 Unique Recombinant Progeny | 2013 | - | 39 |
| AC043 | wildtype | NF54GFP × NHP4026 Unique Recombinant Progeny | 2013 | - | 39 |
| AC049 | wildtype | NF54GFP × NHP4026 Unique Recombinant Progeny | 2013 | - | 39 |
| AC056 | wildtype | NF54GFP × NHP4026 Unique Recombinant Progeny | 2013 | - | 39 |
| AC077 | wildtype | NF54GFP × NHP4026 Unique Recombinant Progeny | 2013 | - | 39 |
| AC098 | wildtype | NF54GFP × NHP4026 Unique Recombinant Progeny | 2013 | - | 39 |
| AC105 | wildtype | NF54GFP × NHP4026 Unique Recombinant Progeny | 2013 | - | 39 |
| AC120 | wildtype | NF54GFP × NHP4026 Unique Recombinant Progeny | 2013 | - | 39 |
| AC127 | wildtype | NF54GFP × NHP4026 Unique Recombinant Progeny | 2013 | - | 39 |
| AC130 | wildtype | NF54GFP × NHP4026 Unique Recombinant Progeny | 2013 | - | 39 |

**Additional file 1. Table of parasites used in study with origin, year isolated, *kelch13* status, PC_1/2_ (if known), GRRA phenotypes, and publication reference.** [30,39,40,44–54]

**
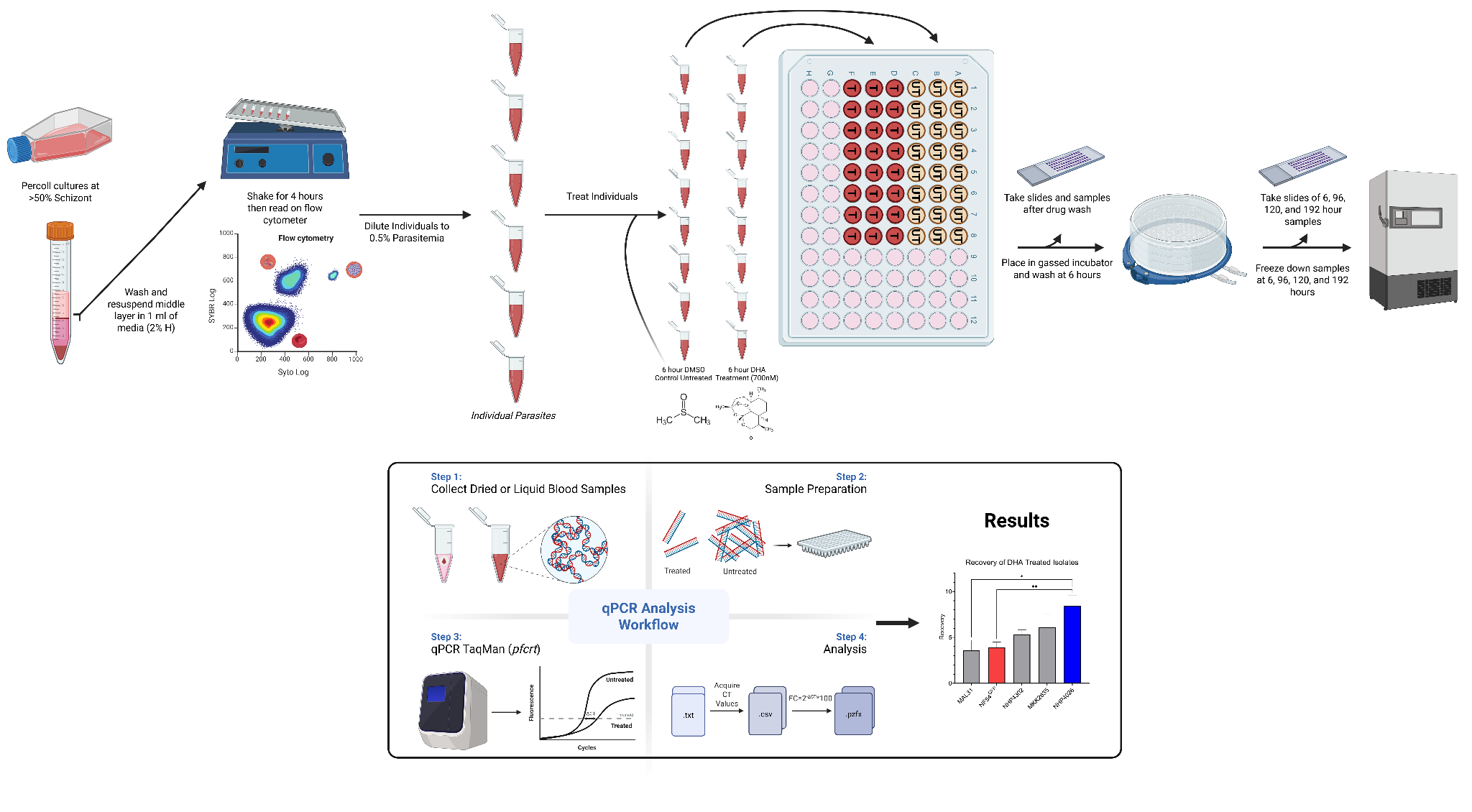
**

**Additional file 2. Schematic of Growth, Resistance, and Recovery Assay** Created in BioRender. Qahash, T. (2025) https://BioRender.com/re5hmuv

| Volume | Reagent | Supplier |
| --- | --- | --- |
| 5 μL | 2X Phusion Blood Direct Mix Buffer | ThermoFisher, cat # F547L |
| 0.25 μL | pfCRT Forward Primer  (AGATTTCGTAACTTTGGTAAGTGTG) | Integrated DNA Technologies |
| 0.25 μL | pfCRT Reverse Primer  (ATGAACGAACAAGCCATTTGAT) | Integrated DNA Technologies |
| 1.4 μL | 7.5X SYBR Green in H_2_O | Invitrogen, cat # S7585 |
| 0.1 μL | Phusion enzyme | ThermoFisher, cat # F547L |
| 3 μL | Thawed culture sample | - |

| Cycle Step | Temp. | Time | Cycle No. |
| --- | --- | --- | --- |
| Initial Denaturation | 95.0°C | 20 s | 1 |
| Denaturation  Annealing  Extension | 95.0°C  62.3°C  65.0°C | 1 s  30 s  15 s | 30 |

**Additional file 3. Table of primers and reagents used for quantitative PCR. Table of cycling conditions used for quantitative PCR.**


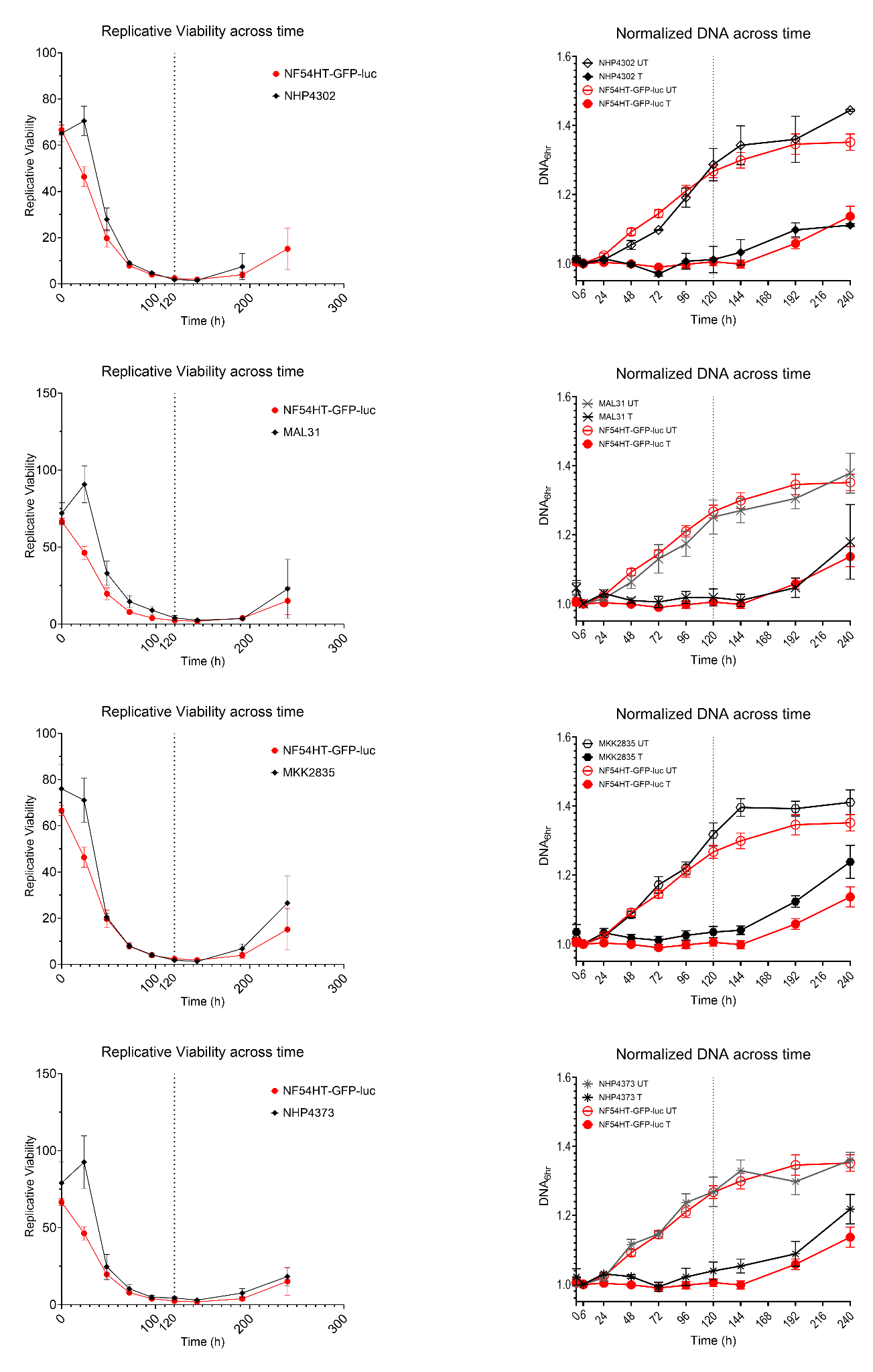


**Additional file 4. Replicative Viability and DNA content of treated and untreated cultures across 10-day time series for additional clinical isolates not shown in main figures.** Each isolate is shown with NF54HT-GFP-luc for reference. Data is reported as mean ± SEM of at least three biological replicates.
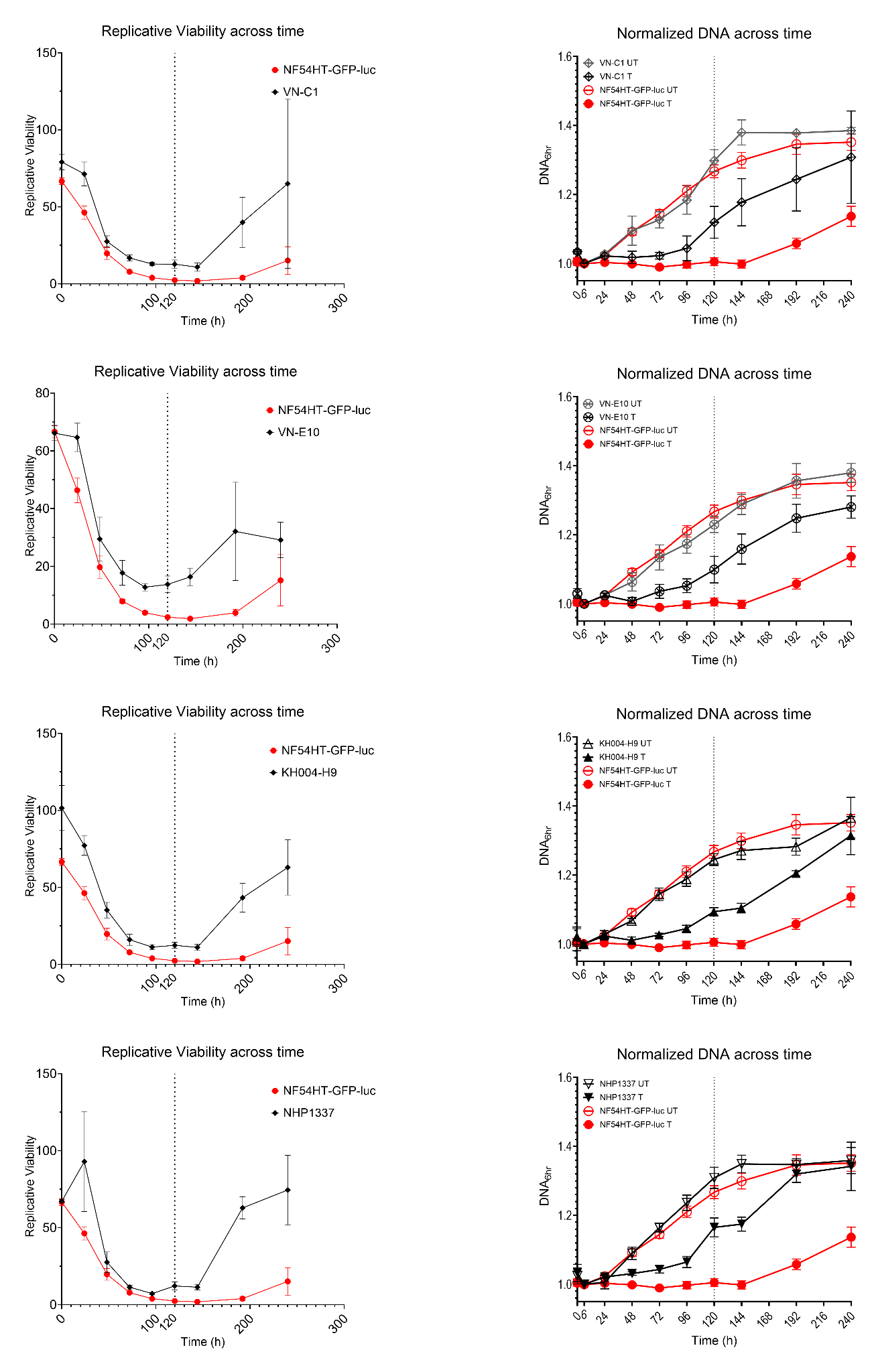


**Additional file 5. Distribution of Resistance (Replicative Viability comparing UT and Treated samples at 120 h) phenotypes for clinical isolates.** Parasites are ordered by increasing level of resistance and include parents of recent genetic crosses (NF54HT-GFP-luc × NHP4026, MKK2835 × NHP1337, MAL31 × KH004-H9), NHP4302 and additional isolates (NHP4373, VN-C1, VN-E10). Parasites with a K13^C580Y^ mutation are marked in black and K13 wildtype parasites are marked in grey. The dashed line at a replicative viability of 4 adapted from the threshold for resistance to correlate with PC_1/2_, as previously reported [27]. Data is reported as mean ± SEM of at least three biological replicates.
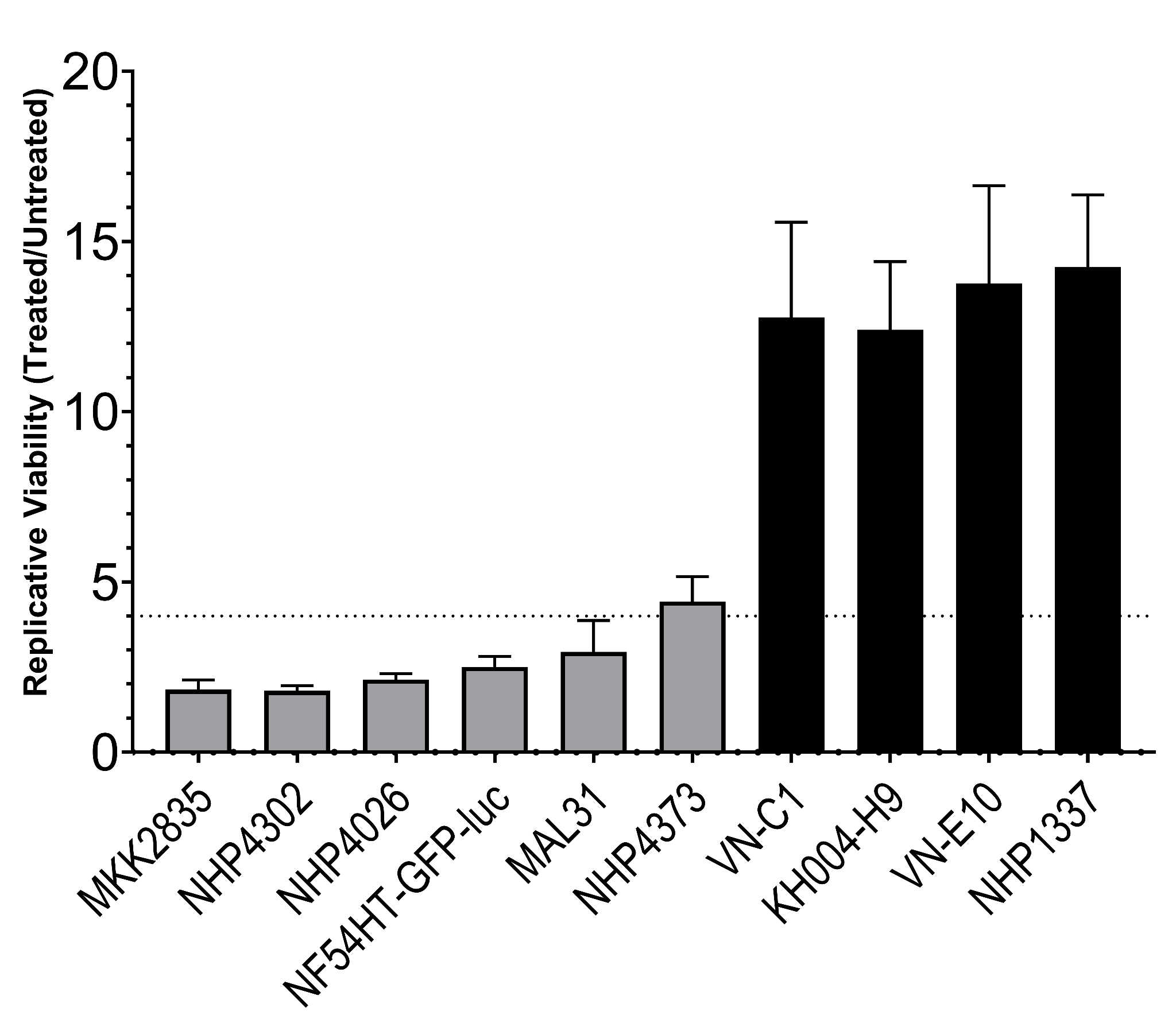


**Additional file 6. Scatterplot of Resistance vs Recovery phenotypes measured in 23 progeny (purple) and parents of the NF54(HT-GFP-luc) (red) × NHP4026 (blue) genetic cross.** Linear regression does not show a significant association Resistance and Recovery (R^2^ = 0.0021, F(1, 23) = 0.0501, *P*-value= 0.8249). The dotted lines denote the 95% confidence interval around the best fit line (*y* = -0.06616*x* + 1.123). Linear regression was performed using mean phenotypes of at least three biological replicates and phenotype values are normalized to the NF54HT-GFP-luc parent.
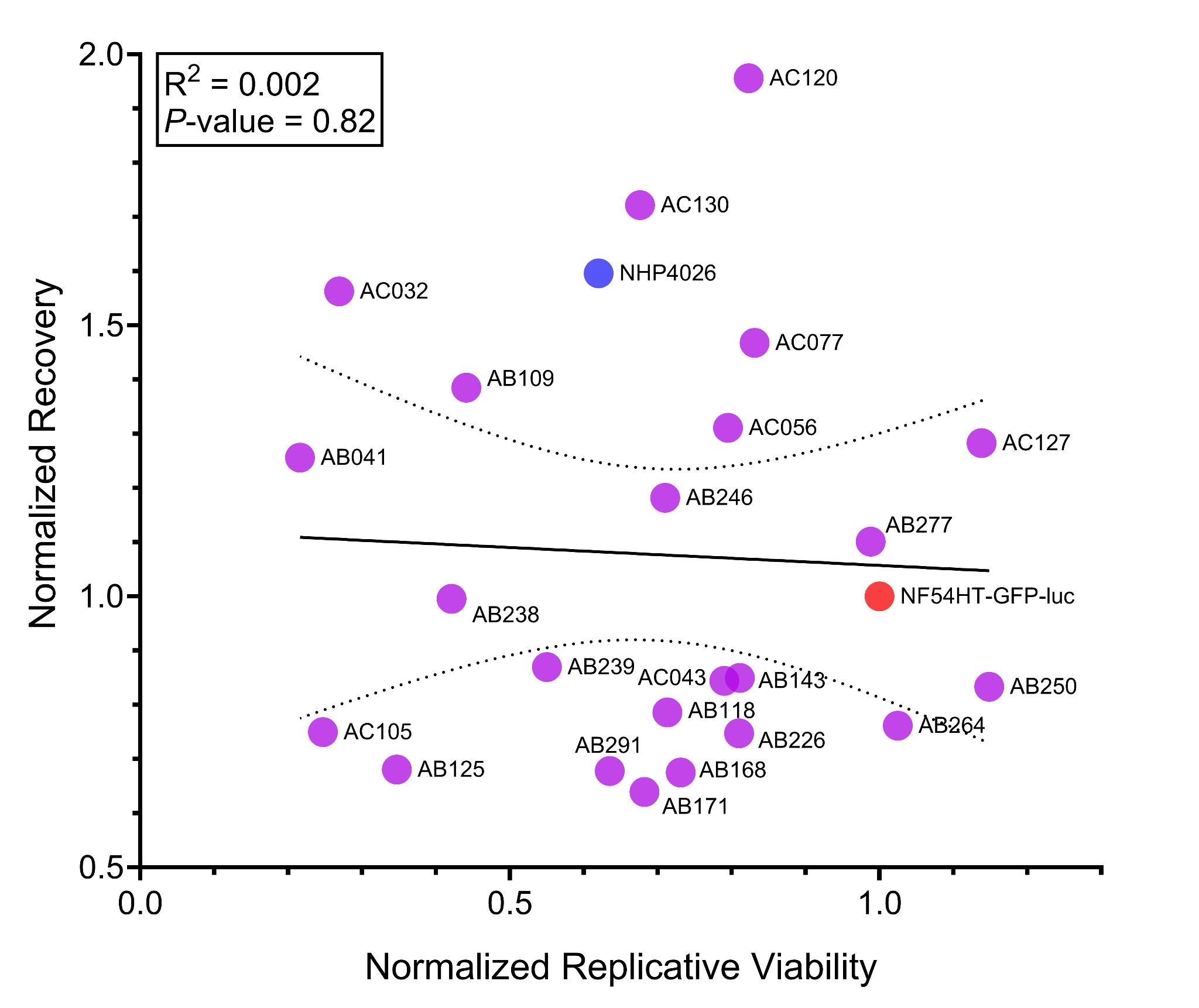


**Additional file 7. Scatterplot of PMR and asexual cell cycle duration measured in eight progeny (grey dots) and parents of the HB3 (yellow) ×** **Dd2 (green) genetic cross.** Linear regression between phenotypes shows a significant linear association that PMR increases as cell cycle duration decreases and 46.6% of variation is shared between the phenotypes (R^2^ = 0.4661, F(1, 8) = 6.984, *P*-value = 0.0296). The dotted lines denote the 95% confidence interval around the best fit line (*y* = -0.3115*x* + 17.3). Linear regression was performed using mean phenotypes of at least three biological replicates or accessed from previously published sources [31].
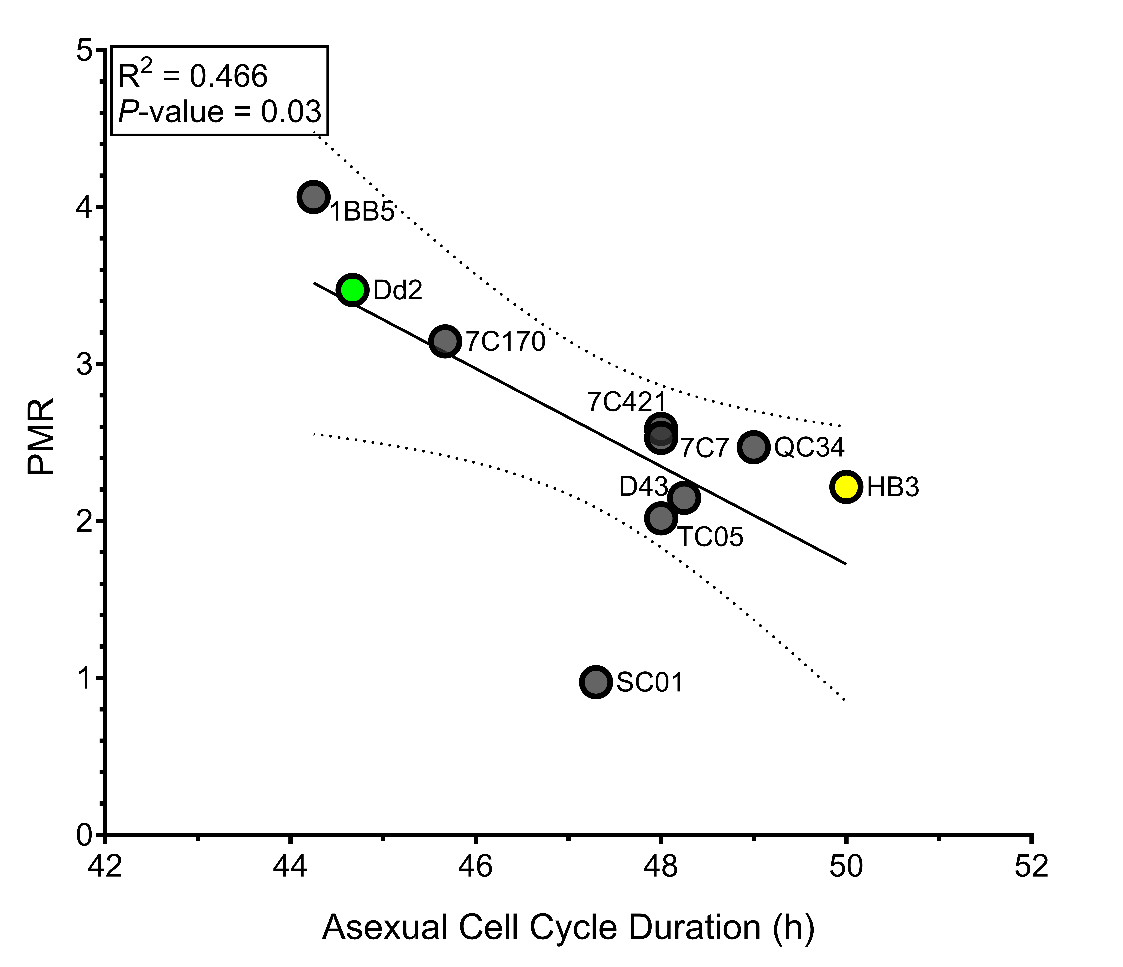


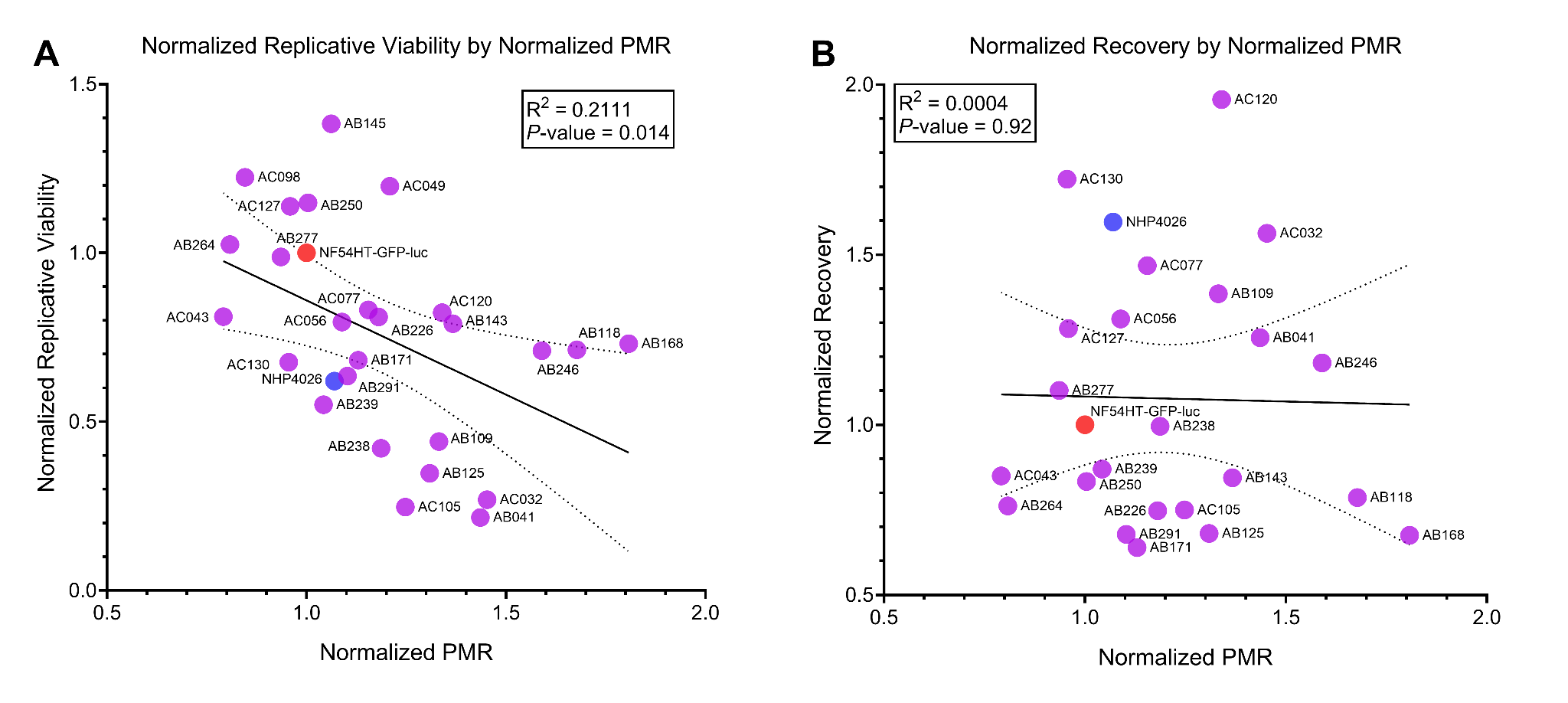
**Additional file 8. Scatterplot of PMR vs Replicative Viability or Recovery phenotypes in progeny (purple dots) and parents of the NF54HT-GFP-luc (red) × NHP4026 (green) genetic cross. A)** Linear regression of PMR vs Replicative Viability shows a significant and positive linear association with 21.1% of variation shared between the phenotypes (R^2^ = 0.2111, F(1, 26) = 6.956, *P*-value= 0.0139). The dotted lines denote the 95% confidence interval around the best fit line (*y* = -0.5582*x* + 1.418). **B)** Linear regression of PMR vs Recovery shows no significant association (R^2^ = 0.0004, F(1, 23) = 0.009, *P*-value= 0.9234). The dotted lines denote the 95% confidence interval around the best fit line (*y* = -0.02936*x* + 1.113). Linear regression was performed using mean phenotypes of at least three biological replicates and phenotype values are normalized to the NF54HT-GFP-luc parent.
